# CmABF3-*CmTPP2/5* modulates trehalose-mediated hydrogen peroxide signaling and CBF-dependent pathway to enhance cold tolerance in melon seedlings

**DOI:** 10.1093/hr/uhag233

**Published:** 2026-06-08

**Authors:** Tao Liu, Hongyi Zhang, Kexin Lu, Jiahui Tian, Yuqing Han, Haiming Li, Yujie Zhang, Lin Dong, Xueling Ye, Hongyan Qi

**Affiliations:** College of Horticulture, Shenyang Agricultural University, Shenyang 110866, China; Key Laboratory of Protected Horticulture of Education of Ministry and Liaoning Province, Northern National & Local Joint Engineering Research Center of Horticultural Facilities Design and Application Technology, Shenyang, Liaoning 110866, China; College of Horticulture, Shenyang Agricultural University, Shenyang 110866, China; Key Laboratory of Protected Horticulture of Education of Ministry and Liaoning Province, Northern National & Local Joint Engineering Research Center of Horticultural Facilities Design and Application Technology, Shenyang, Liaoning 110866, China; College of Horticulture, Shenyang Agricultural University, Shenyang 110866, China; Key Laboratory of Protected Horticulture of Education of Ministry and Liaoning Province, Northern National & Local Joint Engineering Research Center of Horticultural Facilities Design and Application Technology, Shenyang, Liaoning 110866, China; College of Horticulture, Shenyang Agricultural University, Shenyang 110866, China; Key Laboratory of Protected Horticulture of Education of Ministry and Liaoning Province, Northern National & Local Joint Engineering Research Center of Horticultural Facilities Design and Application Technology, Shenyang, Liaoning 110866, China; College of Horticulture, Shenyang Agricultural University, Shenyang 110866, China; Key Laboratory of Protected Horticulture of Education of Ministry and Liaoning Province, Northern National & Local Joint Engineering Research Center of Horticultural Facilities Design and Application Technology, Shenyang, Liaoning 110866, China; College of Horticulture, Shenyang Agricultural University, Shenyang 110866, China; Key Laboratory of Protected Horticulture of Education of Ministry and Liaoning Province, Northern National & Local Joint Engineering Research Center of Horticultural Facilities Design and Application Technology, Shenyang, Liaoning 110866, China; College of Horticulture, Shenyang Agricultural University, Shenyang 110866, China; Key Laboratory of Protected Horticulture of Education of Ministry and Liaoning Province, Northern National & Local Joint Engineering Research Center of Horticultural Facilities Design and Application Technology, Shenyang, Liaoning 110866, China; College of Horticulture, Shenyang Agricultural University, Shenyang 110866, China; Key Laboratory of Protected Horticulture of Education of Ministry and Liaoning Province, Northern National & Local Joint Engineering Research Center of Horticultural Facilities Design and Application Technology, Shenyang, Liaoning 110866, China; College of Horticulture, Shenyang Agricultural University, Shenyang 110866, China; Key Laboratory of Protected Horticulture of Education of Ministry and Liaoning Province, Northern National & Local Joint Engineering Research Center of Horticultural Facilities Design and Application Technology, Shenyang, Liaoning 110866, China; College of Horticulture, Shenyang Agricultural University, Shenyang 110866, China; Key Laboratory of Protected Horticulture of Education of Ministry and Liaoning Province, Northern National & Local Joint Engineering Research Center of Horticultural Facilities Design and Application Technology, Shenyang, Liaoning 110866, China

## Abstract

Cold stress inhibits the growth and development of melon. Exogenous trehalose (Tre) has been reported to alleviate cold damage in melon seedlings; however, it remains unclear whether plants respond to cold stress by activating endogenous Tre synthesis and the associated upstream and downstream pathways. Here, the results show that cold stress markedly increases endogenous Tre levels in melon leaves. Transcriptome analysis identified two trehalose-6-phosphate phosphatase (key enzymes in Tre synthesis, TPP) genes, *CmTPP2* and *CmTPP5*, which were strongly induced by cold stress. Silencing *CmTPP2/5* significantly reduced Tre content, lowered the activities of superoxide dismutase, ascorbate peroxidase and glutathione reductase, and exacerbated cold-induced lipid peroxidation damage; meanwhile, it inhibited apoplastic H_2_O_2_ accumulation, restricted H_2_O_2_ transport into the cytoplasm, and downregulated the expression of C-repeat binding factor 1/2 (*CmCBF1/2*) under cold stress. Protein–DNA interaction assays demonstrated that the transcription factor CmABF3 binds to the ABRE motif in the *CmTPP2/5* promoter to activate their transcription. Silencing *CmABF3* gene significantly reduced *CmCBF1*/2 and *CmTPP2/5* expression and reduced Tre accumulation. Inhibition of trehalase activity by validamycin A elevated Tre content, enhanced antioxidant capacity under cold stress, promoted apoplastic H_2_O_2_ production and transport, and upregulated *CmCBF1/2* expression. Silencing *CmCBF1/2* also inhibited the production and transport of apoplastic H₂O₂ and reduced melon cold tolerance. These findings reveal that cold stress triggers CmABF3-mediated activation of *CmTPP2/5*, leading to Tre accumulation, which then enhanced antioxidant defenses via regulation of apoplastic H_2_O_2_ production and transport, and coordination with the CBF-dependent pathway to improve cold tolerance in melon seedlings.

## Introduction

Cold stress limits normal plant growth and development, thereby reducing crop yield and quality [1–3]. When exposed to cold stress, plant cells sense and respond to low temperatures through changes in cell membrane fluidity. These signals are then transmitted by cytoplasmic signaling molecules, such as calcium (Ca^2+^), reactive oxygen species (ROS), and plant hormones [4], and further modulated by various kinases and transcription factors to activate downstream pathways [such as inducer of CBF expression (ICE)- C-repeat binding factor (CBF)- cold-regulated gene (COR)], ultimately enhancing cold tolerance [5].

Apoplastic hydrogen peroxide (H_2_O_2_), a kind of stable ROS, primarily produced by nicotinamide adenine dinucleotide phosphate (NADPH) oxidases, acts as a key early signaling molecule in plant stress responses. Evidence shows that apoplastic H_2_O_2_ signaling enhances disease resistance by activating immune pathways [6–8]. It also contributes to adaptation to abiotic stresses, such as drought [9, 10], salinity [11, 12], and heat stress [13, 14]. During cold stress, SlRBOH1-mediated apoplastic H_2_O_2_ enhances tomato antioxidant capacity by regulating glutathione (GSH) synthesis [15] and glutaredoxin levels [16]. H_2_O_2_ also acts synergistically with abscisic acid (ABA) [4, 17], Ca^2+^ [18], and brassinosteroids [19] to improve cold tolerance. Therefore, apoplastic H_2_O_2_ is central to stress perception and the activation of downstream antioxidant defenses.

However, prolonged or severe cold stress can cause excessive H_2_O_2_ accumulation, causing oxidative damage and programmed cell death [20]. To maintain redox homeostasis, plants regulate both the generation of H_2_O_2_ and the activity of antioxidant systems. These systems include enzymatic components—superoxide dismutase (SOD), peroxidase (POD), catalase, ascorbate peroxidase (APX), glutathione reductase (GR)—and nonenzymatic components, such as ascorbic acid (AsA) and GSH [21]. Enhancing antioxidant defenses under adverse conditions is therefore essential for maintaining ROS balance and sustaining growth.

Trehalose (Tre) is a nonreducing disaccharide present in many organisms and is widely reported to enhance plant stress tolerance by regulating antioxidant enzyme activity, protecting cell membranes from oxidative damage, and maintaining osmotic balance under stresses including drought, salinity, cold, and heat [22–25]. In plants, Tre is synthesized from uridine diphosphate glucose and glucose-6-phosphate via the sequential action of trehalose-6-phosphate synthase (TPS) and trehalose-6-phosphate phosphatase (TPP), and can be hydrolyzed into two glucose molecules by trehalase [26]. Tre functions as an osmoprotectant [27, 28], stabilizing dehydrated enzymes and proteins, and protecting biological systems from abiotic stress [29]. Exogenous Tre application can also act as a stimulus, inducing stress signaling, modulating downstream responses, and regulating stress-related genes expression to improve tolerance [30–32].

Previous research demonstrated that foliar Tre application enhances antioxidant capacity and significantly improves the cold tolerance of melon seedlings [22, 25, 33]. Nevertheless, the role of endogenous Tre in low-temperature responses and its regulatory mechanisms in plant cold tolerance remain unclear. This study aims to elucidate the role of the final key biosynthetic enzyme, TPP, in Tre regulation and its contribution to cold tolerance in melon.

TPP is widely distributed in higher plants, and the *TPP* gene family has been identified in multiple species [34]. *TPP* has been shown to participate in plant responses and resistance to various abiotic stresses. In cotton, silencing of the *GhTPP22* gene under drought stress reduced stomatal closure and antioxidant capacity, making plants more sensitive to drought [35]. Overexpression of a *TPS*–*TPP* fusion gene in transgenic rice leaves and seeds increased Tre content, thereby enhancing tolerance to drought, salinity, and low temperature [36]. Similarly, overexpression of *OsTPP3* in rice [37] and *TPP* genes in maize [38] improved drought tolerance. In *Arabidopsis*, low temperature induced the expression of *AtTPPI*, leading to increased Tre accumulation and enhanced cold tolerance [39]. These findings demonstrate that TPP-mediated Tre synthesis plays a critical role in regulating plant stress responses and resistance. However, the precise molecular mechanisms remain unclear.

Under stress conditions, *TPPs* gene expression is regulated by diverse transcription factors. In *Arabidopsis*, cold stress-induced CBF1/2/3 directly regulate *AtTPPI* expression, promoting Tre synthesis and enhancing cold tolerance [39]. DREB1A binds to the *AtTPPF* promoter to activate its expression, increasing soluble sugar accumulation and protecting seedling cells from ROS-induced damage under drought stress [40]. In rice, OsBHLH002/OsICE1 is phosphorylated by the mitogen-activated protein kinase OsMAPK3 and activates downstream *OsTPP1*, increasing Tre content and improving low-temperature tolerance [41]. Additionally, low temperature-induced OsMYB30 directly regulates *OsTPP1*, promoting Tre accumulation in rice seeds and inhibiting seed germination [42]. Despite these advances, the functions and mechanisms of melon *CmTPPs* genes in stress responses and tolerance regulation remain unknown.

Melon is an important global economic crop, preferring warm climates and showing high sensitivity to cold stress. Cold damage restricts growth and development, delays market availability, and causes substantial economic losses. This study investigates the function and mechanism of endogenous Tre synthesis catalyzed by TPP in melon responses to cold stress and in cold tolerance regulation. Cold stress strongly induces the ABA-responsive transcription factor CmABF3. Protein–DNA interaction assays confirmed that CmABF3 binds to the promoter region of *CmTPP2/5*, activating their expression and increasing endogenous Tre levels. The elevated Tre enhances apoplastic H_2_O_2_ production and transport, triggering redox signaling and enhancing antioxidant defenses. Concurrently, Tre mediates activation of the CBF-dependent pathway, jointly improving cold tolerance in melon seedlings.

In conclusion, the CmABF3-*CmTPP2/5* transcriptional regulatory module synergistically enhances melon cold tolerance by positively regulating both the H_2_O_2_ signaling and the CBF-dependent pathway. These findings provide candidate genes for cold tolerance breeding in melon.

## Results

### Cold stress induces trehalose accumulation and upregulates *CmTPPs* expression in melon seedlings

Tre plays an important role in plant stress responses and tolerance [25]. In this study, Tre content in melon leaves increased significantly after cold treatment (Fig. 1a), suggesting a role in the regulation of cold response and tolerance in melon seedlings. Therefore, the response of TPP, a key rate-limiting enzyme in Tre synthesis, to cold stress was further investigated.

**Figure 1:**
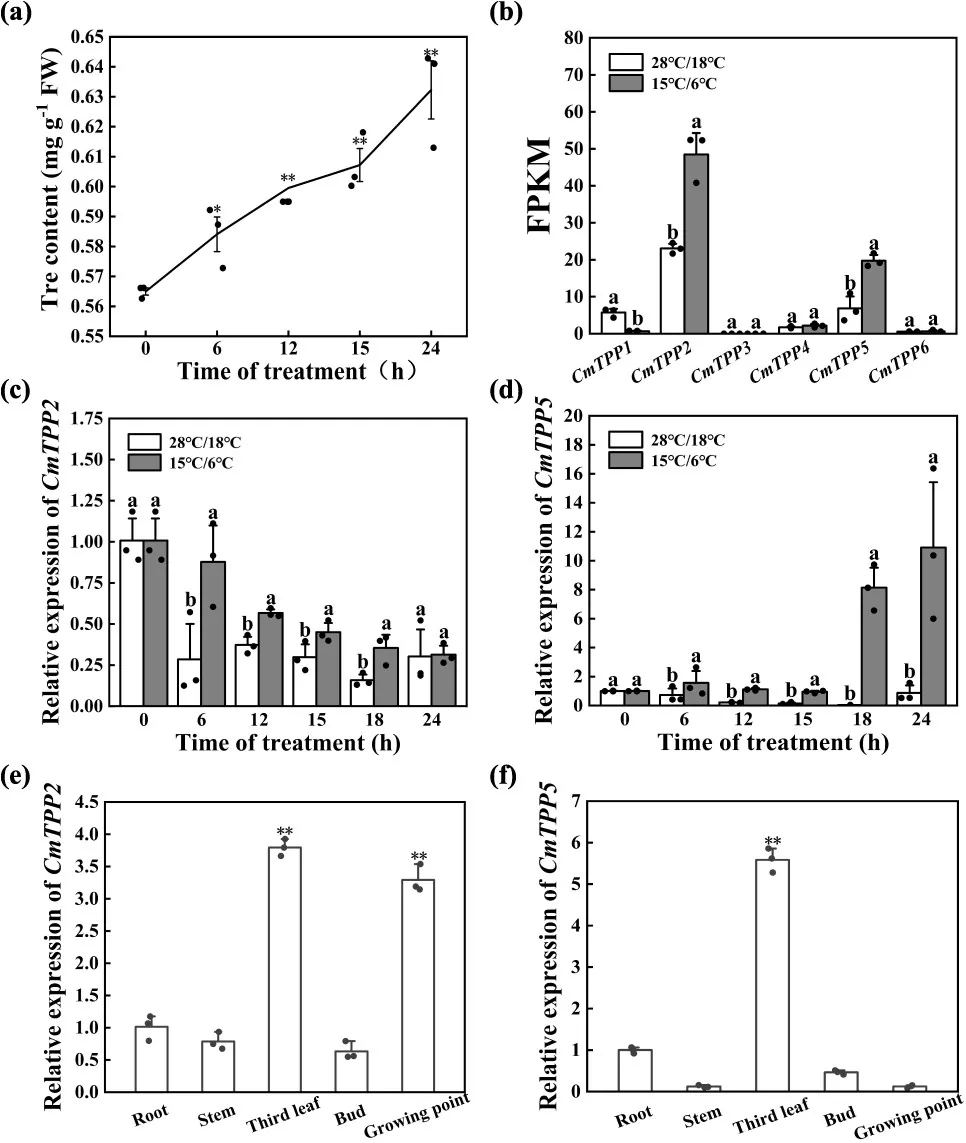
Cold stress elevates Tre content and *CmTPP2/5* expression in melon seedlings. (a) Tre content within 24 h after cold stress treatment (15°C/6°C, day/night). (b) Transcriptome analysis of *CmTPPs* in melon seedlings under normal conditions (28°C/18°C, day/night) or cold treatment (15°C/6°C, day/night) for 18 h. (c and d) Analyzation of the *CmTPP2*/*5* expression using RT-qPCR under normal condition or cold stress treatment for 24 h. The *CmTPPs* expression in melon seedlings under normal condition at 0 h were normalized as 1. (e and f) Analyzation of the *CmTPP2*/*5* expression in different tissues of melon using RT-qPCR at the same time. The *CmTPPs* expression in root were normalized as 1. Data are the means ± standard error of three biological replicates. Different letters or * indicate a significant difference of *P* <0 .05, while ^**^ represent a significant difference at *P* <0 .01 according to Duncan’s multiple range test.

Based on reference sequences from *Arabidopsis thaliana*, six *CmTPPs* genes were identified from the Cucurbit genome database. Their protein sequences were retrieved, and a phylogenetic tree was constructed using MEGA 11, showing that the *CmTPPs* family can be classified into three groups (Fig. S1a). A chromosome location map was generated according to the genomic positions of the six genes (Fig. S1b), which were designated as *CmTPP1* (MELO3C011362.2.1), *CmTPP2* (MELO3C006812.2.1), *CmTPP3* (MELO3C006984.2.1), *CmTPP4* (MELO3C013969.2.1), CmTPP5 (MELO3C017588.2.1), and *CmTPP6* (MELO3C007768.2.1).

Transcriptome analysis revealed that cold treatment significantly upregulated *CmTPP2* and *CmTPP5* expression and reduced *CmTPP1* expression, and had no significant influence on *CmTPP3*, *CmTPP4*, and *CmTPP6* expression, compared to normal temperature (Fig. 1b).

Quantitative real-time polymerase chain reaction (RT-qPCR) analysis confirmed these trends, showing that cold stress significantly induced *CmTPP2* and *CmTPP5* expression (Fig. 1c and d), while tissue-specific expression profiling showed that *CmTPP2* and *CmTPP5* had the highest expression levels in the third leaf (Fig. 1e and f; Fig. S1c and d). Therefore, these two genes were therefore selected for subsequent functional studies.

### *CmTPP2/5* positively regulate cold tolerance in melon seedlings

To clarify the roles of *CmTPP2* and *CmTPP5* in cold stress response and tolerance, virus-induced gene silencing (VIGS) was used to generate pV190-*CmTPP2* and pV190-*CmTPP5* silenced lines. The pV190-*CmPDS* line displayed a clear albino phenotype (Fig. S2a and b), and the silencing efficiency of pV190-*CmTPP2* and pV190-*CmTPP5* reached 50% and 60%, respectively, compared with the pV190 control line (Fig. S2c and d). These results indicate that the *CmTPP2*- and *CmTPP5*-silenced lines were successfully established for functional assays.

To further evaluate cold tolerance, physiological indices were compared. Under normal temperature, phenotypes, relative electrical conductivity (REC), and malondialdehyde (MDA) levels did not differ significantly between silenced and control lines (Fig. 2). However, under cold stress, the silenced lines exhibited more severe leaf damage and wilting than the control (Fig. 2a and b), accompanied by significantly higher REC and MDA levels (Fig. 2c–f). These results indicate that silencing *CmTPP2/5* aggravated membrane lipid peroxidation damage under cold conditions.

**Figure 2:**
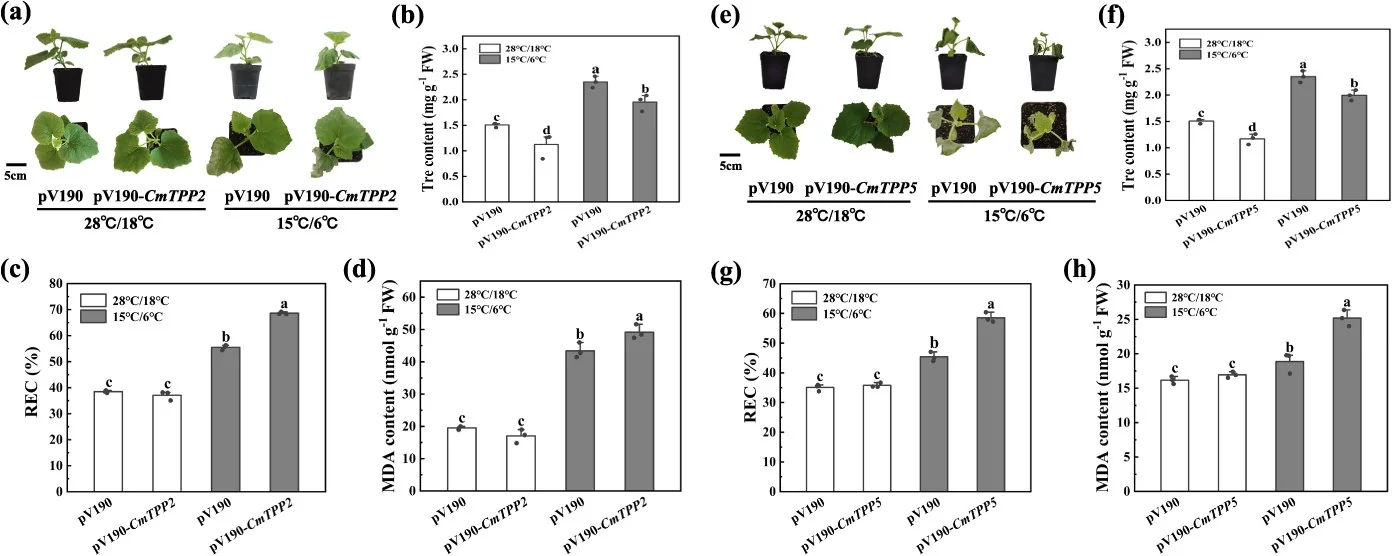
Roles of *CmTPP2/5* in regulating the cold tolerance of melon seedlings. (a and e) Phenotype of pV190 and pV190-*CmTPP2/5* silenced melon seedlings after 24 h of normal temperature (28°C/18°C, day/night) or cold (15°C/6°C, day/night) treatment. (b and f) Tre content, (c and g) REC, (d and h) MDA content in the pV190 and pV190-*CmTPP2/5* silenced melon seedlings under normal condition (28°C/18°C, day/night) or cold stress (15°C/6°C, day/night) treatment for 24 h. Data are the means ± standard error of three biological replicates. Different letters indicate a significant difference of *P* <0 .05 according to Duncan’s multiple range test.

TPP is a key enzyme in Tre synthesis, and cold stress can induce endogenous Tre accumulation. To investigate the role of TPP in Tre synthesis under cold stress, Tre content was measured in pV190 and pV190-*CmTPP2/5* lines after 24 h of cold treatment (Fig. 2g and h). Under the same conditions, Tre content in pV190-*CmTPP2/5* lines was significantly lower than in pV190, although Tre levels in all lines increased under cold stress compared with normal temperature.

Antioxidant capacity reflects a plant’s ability to withstand stress. Under normal temperature, ferric reducing antioxidant power (FRAP) did not differ between pV190 and pV190-*CmTPP2/5* lines. Under cold stress, FRAP increased compared to normal conditions, but the FRAP in silenced lines was significantly lower than in pV190 (Fig. 3a and e). Enzyme activity assays showed no significant differences in SOD, APX, and GR activities between lines under normal temperature. However, under cold conditions, these activities increased compared to normal conditions but were significantly lower in silenced lines than in pV190 (Fig. 3). Treatment with the trehalase inhibitor validamycin A (Val A) reduced REC and MDA levels, while increasing Tre content and the activities of SOD, APX, and GR (Figs S3 and S4). These findings demonstrate that *CmTPP2*- and *CmTPP5*-mediated Tre enhances antioxidant capacity in melon seedlings by elevating SOD, APX, and GR activities (Fig. 3).

**Figure 3:**
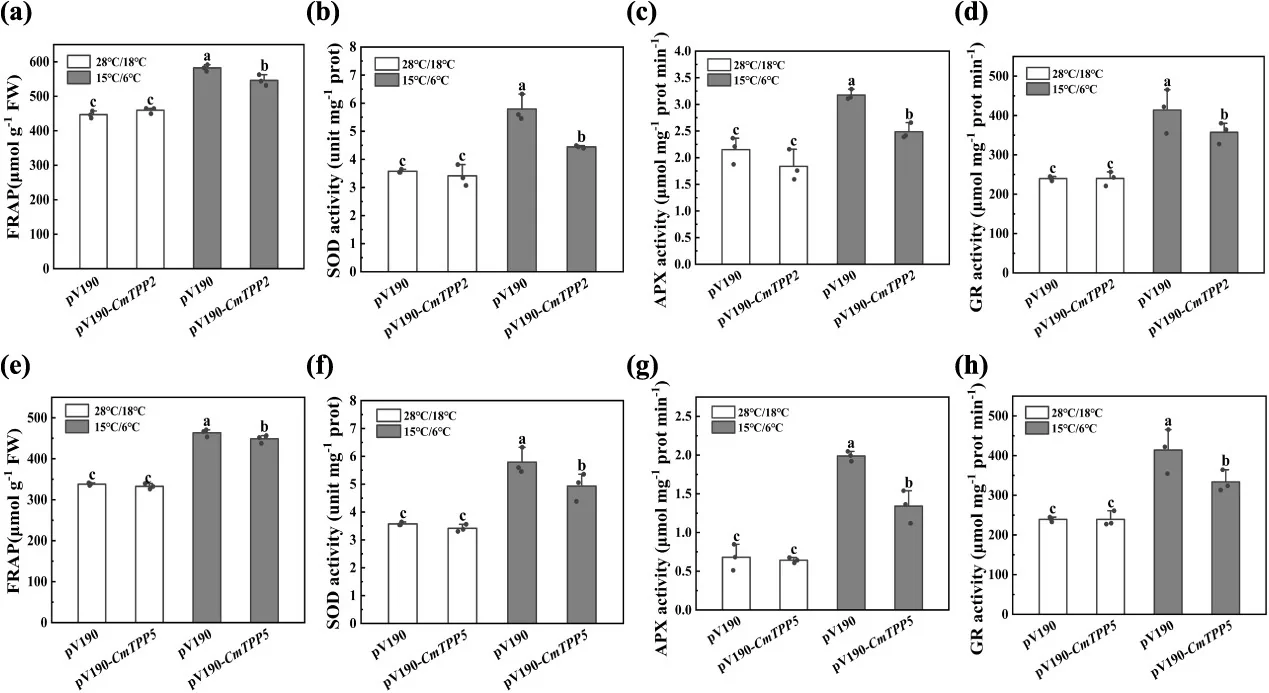
Roles of *CmTPP2/5* in regulating the antioxidation of melon seedlings under cold stress. (a and e) The FRAP, (b and f) SOD activity, (c and g) APX activity, (d and h) GR activity in pV190 and pV190-*CmTPP2/5* silenced melon seedlings after 24 h of normal temperature (28°C/18°C, day/night) or cold (15°C/6°C, day/night) treatment. Data are the means ± standard error of three biological replicates. Different letters indicate a significant difference of *P* <0 .05 according to Duncan’s multiple range test.

### *CmTPP2/5* positively regulate apoplastic hydrogen peroxide production and transport, and *CmCBFs* genes expression in melon seedlings

Previous work demonstrated that apoplastic H_2_O_2_ plays a positive role in enhancing melon cold tolerance [22]. However, whether *CmTPP2/5*-mediated Tre is associated with the apoplastic H_2_O_2_ signal remains unclear. Here, the confocal laser microscopy was used to detect apoplastic H_2_O_2_ fluorescence (Amplex Ultra Red, AUR) and cytoplasmic H_2_O_2_ fluorescence (Amplex Red, AR) in pV190 and pV190-*CmTPP2/5* melon lines under normal temperature [22]. These dyes produce red fluorescence following oxidation with H_2_O_2_ (Fig. 4a). All testing was deliberately carried out at room temperature. This design was necessary owing to the fact that chilling compromises plant cell membrane integrity, which would lead to nonselective dye penetration. Compared with pV190, pV190-*CmTPP2/5* plants exhibited a significantly higher apoplastic H_2_O_2_ signal and a lower cytoplasmic H_2_O_2_ signal (Fig. 4a). The total fluorescence (AUR + AR) was significantly reduced, while the AUR/AR ratio was significantly increased in pV190-*CmTPP2/5* (Fig. 4b and c). These results indicate that *CmTPP2/5*-mediated Tre enhances apoplastic H_2_O_2_ production and its transport into the cytoplasm. Treatment with Val A significantly increased H_2_O_2_ levels and total fluorescence (AUR + AR), while decreasing the AUR/AR ratio (Fig. S5).

**Figure 4:**
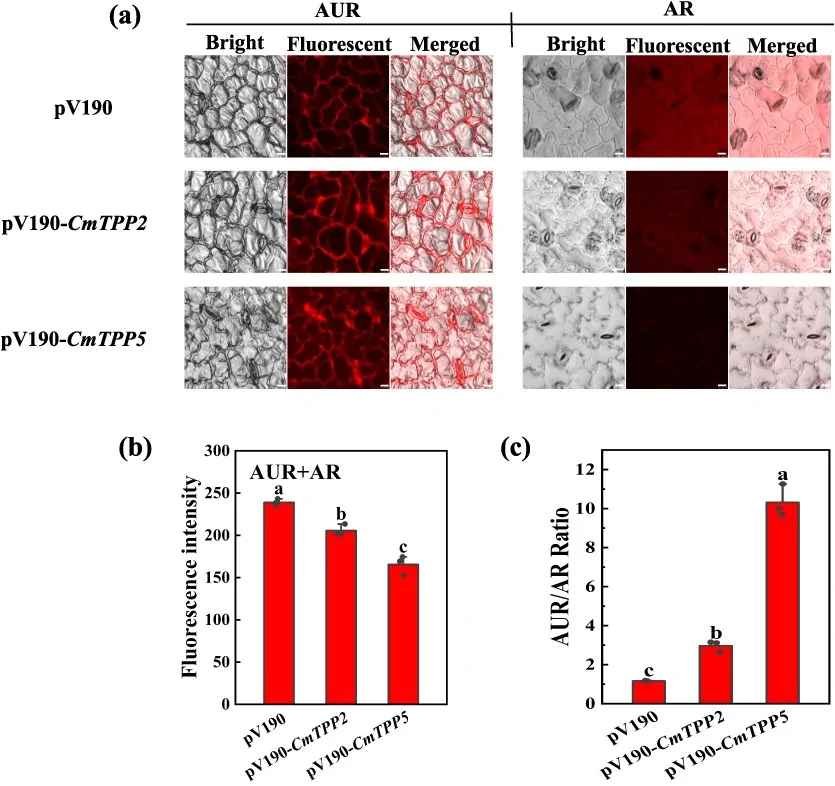
Roles of *CmTPP2* and *CmTPP5* in regulating H_2_O_2_ production and transport in melon seedlings. (a) Imaging of apoplastic and cytoplasmic H_2_O_2_ levels using AUR or AR staining. Scale bars: 10 μm. (b) Fluorescence intensity of AUR + AR and (c) AUR/AR ratio from (a), representing apoplastic and cytoplasmic H_2_O_2_ levels in pV190 and pV190-*CmTPP2/5* silenced seedlings before cold treatment. Data are the means ± standard error of three biological replicates. Different letters indicate a significant difference of *P* <0 .05 according to Duncan’s multiple range test.

Previous studies showed that *CmCBF1*/2/4 play crucial roles in melon cold stress responses [43]. To assess whether *CmTPP2/5* regulate cold tolerance via the CBF-dependent pathway, the *CmCBFs* expression levels was analyzed. Under normal temperature, no significant differences in *CmCBF1*/2/4 expression were observed between pV190 and pV190-*CmTPP2/5* (Fig. 5). Under cold conditions, *CmCBF1* and *CmCBF2* expression was significantly reduced in pV190-*CmTPP2/5* compared with pV190, whereas *CmCBF4* expression remained unchanged (Fig. 5). Subsequent experiments therefore focused on *CmCBF1* and *CmCBF2*. Val A treatment significantly increased *CmCBF1* and *CmCBF2* expression (Fig. S4f and g). In addition, results showed that silencing *CmCBF1* and *CmCBF2* led to a significant increase in REC and MDA contents in melon seedlings, as well as aggravated plant wilting, while treating with Tre partially alleviated the damage caused by silencing CmCBF1/2, indicating that *CmCBF1/2* genes positively regulate cold tolerance in melon seedlings and which partially involved in Tre’s roles (Figs S6 and S7). Silencing *CmCBF1* and *CmCBF2* reduced H_2_O_2_ levels and inhibited the transport of apoplastic H_2_O_2_ into the cell. In contrast, Tre application significantly alleviated the above trends and promoted both the production of apoplastic H_2_O_2_ and its transport into the cytoplasm (Fig. S8). These results suggest that *CmTPP2/5*-Tre may mediate the CmCBF1/2 to regulate the production and transport of apoplastic H_2_O_2_, which then enhanced the cold tolerance in melon seedlings.

**Figure 5:**
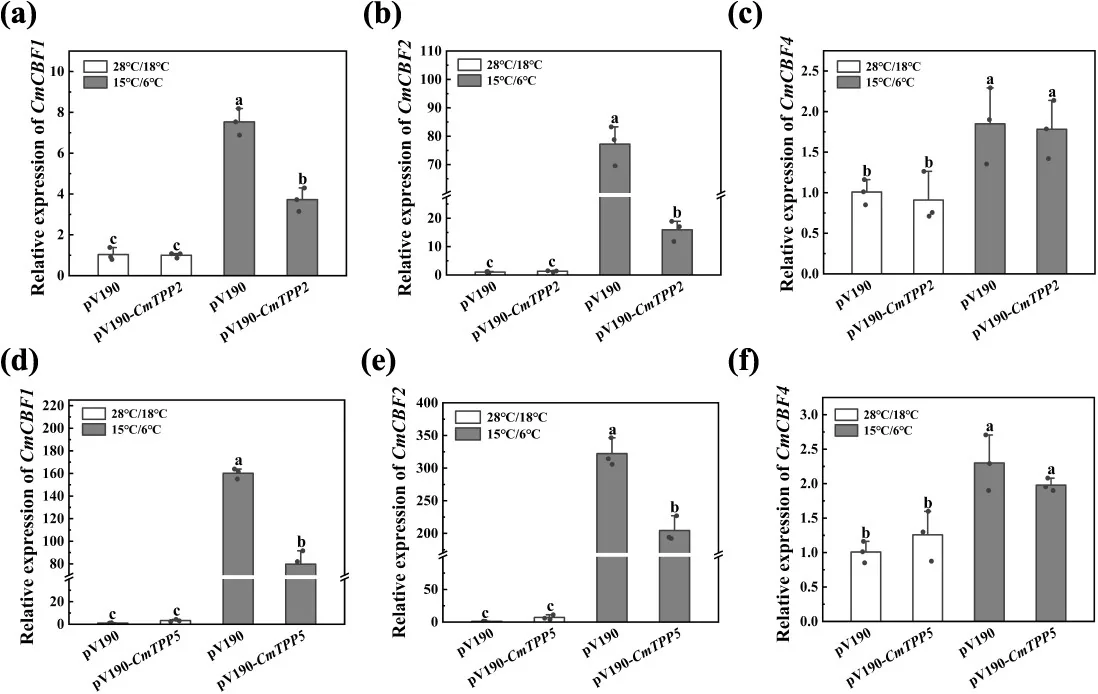
Roles of *CmTPP2/5* in regulating the *CmCBFs* expression in melon seedlings. (a–c) The *CmCBF1/2/4* expression in pV190 and pV190-*CmTPP2* silenced melon seedlings under normal condition (28°C/18°C, day/night) or cold stress (15°C/6°C, day/night) treatment for 24 h. (d–f) The *CmCBF1/2/4* expression in pV190 and pV190-*CmTPP5* silenced melon seedlings under normal condition or cold stress treatment for 24 h. The *CmCBFs* expression in pV190 melon seedlings under normal condition were normalized as 1. Data are the means ± standard error of three biological replicates. Different letters indicate a significant difference of *P* <0 .05 according to Duncan’s multiple range test.

### CmABFs positively regulate *CmTPP2/5* transcription

To identify upstream transcriptional regulators of *CmTPP2/5*, the 2000 bp promoter regions upstream of their transcription start sites was analyzed using PlantCARE (http://bioinformatics.psb.ugent.be/webtools/plantcare/html/). Both promoters contained ABRE elements, which are binding sites for ABF transcription factors (Fig. 6a and b). Previous work indicated that *CmABF2* and *CmABF3* positively regulate melon cold tolerance [22]. We therefore examined whether these transcription factors interact with *CmTPP2/5* promoters.

**Figure 6:**
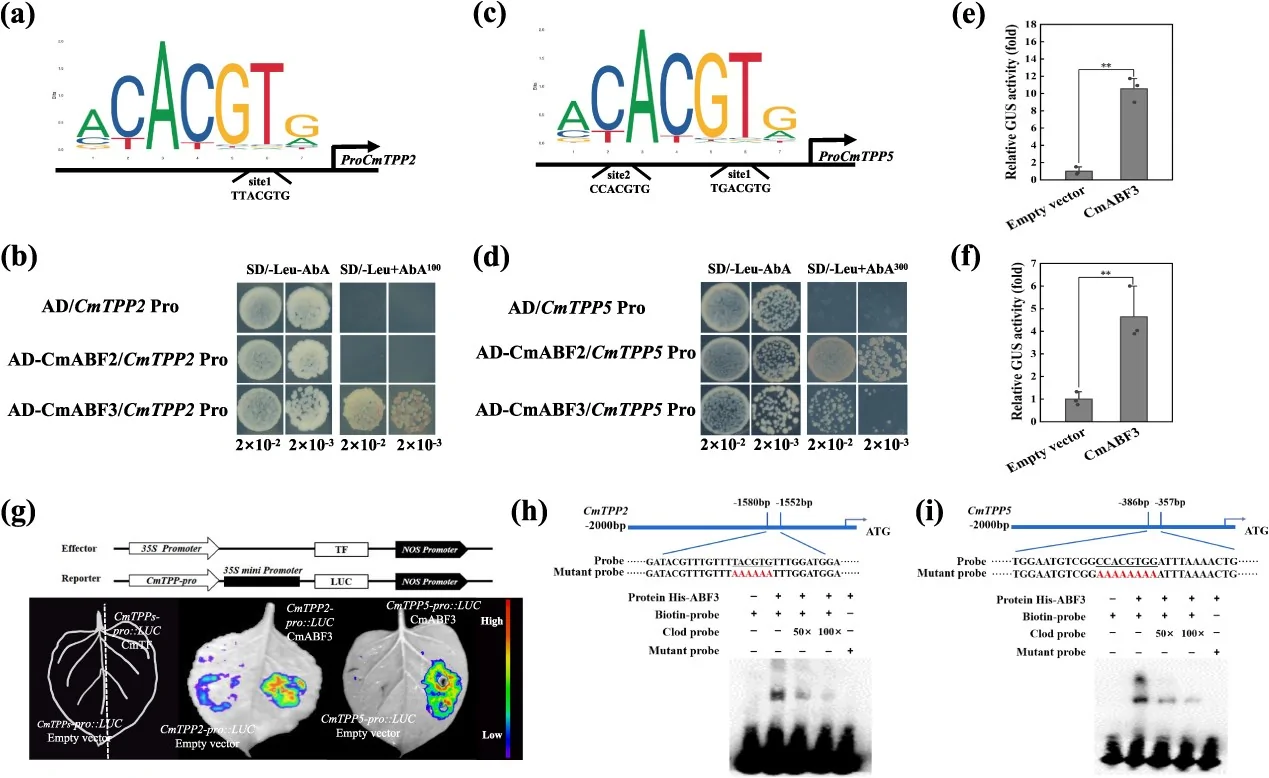
Transcriptional regulation of *CmTPP2/5* by CmABF3. (a–d) Y1H assay detecting CmABF2/3 binding to the *CmTPP2/5* promoter fragment. (e and f) GUS activity assay showing CmABF3 binding to the *CmTPP2/5* promoter enhances promoter activity. (g) Luciferase reporter assay demonstrating CmABF3-mediated activation of *CmTPP2/5* promoters. (h and i) EMSA assays showing that CmABF3 proteins directly bind to the biotin-labeled probe of the *CmTPP2/5* promoter fragment. Data are the means ± standard error of three biological replicates. ** represent a significant difference at *P* <0 .01 according to Duncan’s multiple range test.

Yeast one-hybrid (Y1H) assays showed that yeast containing AD-CmABF2 and the *CmTPP2* promoter failed to grow on SD/−Leu medium with 100 ng·ml^–1^ AbA, whereas AD-*CmABF3* with the *CmTPP2* promoter grew well. Both AD-CmABF2 and AD-CmABF3 grew with the *CmTPP5* promoter at 300 ng·ml^–1^ AbA. No growth was observed in yeast with empty AD vectors and *CmTPP2/5* promoters. These findings indicate that CmABF2 interacts with the *CmTPP5* promoter, while *CmABF3* interacts with both *CmTPP2* and *CmTPP5* promoters in yeast (Fig. 6c and d). Given their similar functional roles, *CmABF3*—which can bind both promoters—was selected for subsequent transcriptional regulation experiments.

β-Glucuronidase (GUS) activity assays in tobacco leaves showed that co-infiltration of 35S::*CmABF3* with *CmTPP2*-pro::GUS increased GUS activity ~10-fold relative to the empty vector control, while co-infiltration with *CmTPP5*-pro::GUS increased activity ~4.5-fold (Fig. 6e and f). These results demonstrate that *CmABF3* binds *CmTPP2/5* promoters *in vivo* and activates their expression. Luciferase (LUC) assays confirmed this, with tobacco leaves expressing CmABF3 showing significantly stronger fluorescence signals than controls (Fig. 6g). Electrophoretic mobility shift assay (EMSA) results corroborated that His-tagged CmABF3 protein directly bound the *CmTPP2/5* promoter *in vitro* (Fig. 6h and i).

### *CmABF3* positively regulates trehalose content and *CmCBF1*/2 expression in melon seedlings

To determine whether *CmABF3* affects *CmCBF1*/2 expression and endogenous Tre content, VIGS was used to generate the pV190-*CmABF3* silenced line. The pV190-*CmPDS* positive control exhibited a white phenotype (Fig. 7a). Using the pV190 empty vector line as a reference, the silencing efficiency of pV190-*CmABF3* reached 60% (Fig. 7b), confirming successful gene silencing.

**Figure 7:**
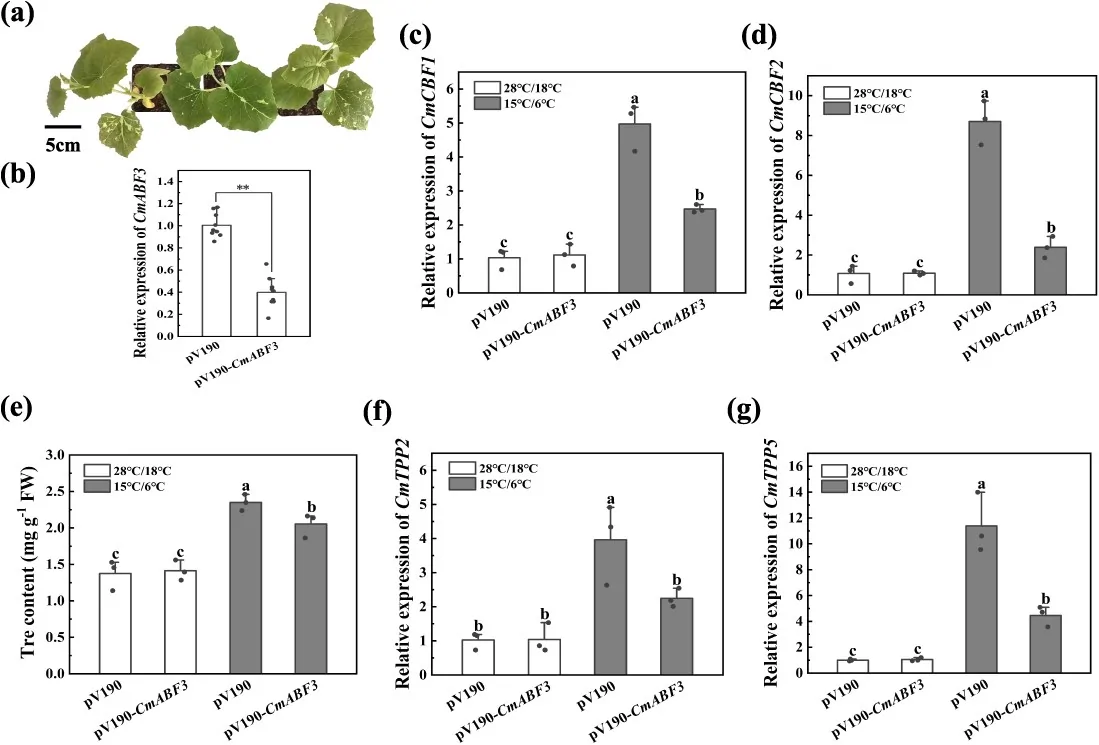
Roles of *CmABF3* in regulating Tre content, *CmCBF1/2* and *CmTPP2/5* expression under cold stress in melon seedlings. (a) The phenotype of pV190-*CmPDS* melon seedlings, indicating VIGS system efficiency. (b) Expression of *CmABF3* in pV190 and pV190-*CmABF3* silenced melon seedlings under normal temperature (28°C/18°C, day/night). The *CmABF3* expression in pV190 plants were normalized as 1. (c and d) The *CmCBF1/2* expression, (e) Tre content, (f and g) The expression of *CmTPP2/5* in pV190 and pV190-*CmABF3* silenced melon seedlings under normal condition (28°C/18°C, day/night) or cold stress (15°C/6°C, day/night) treatment for 24 h. Data are the means ± standard error of three or nine biological replicates. Different letters indicate a significant difference of *P* <0 .05, while ^**^ represent a significant difference at *P* <0 .01 according to Duncan’s multiple range test.

Under normal temperature, no significant differences in *CmCBF1* or *CmCBF2* expression were observed between pV190-*CmABF3* and pV190. Under cold conditions, both lines exhibited significantly higher *CmCBF1* and *CmCBF2* expression compared with normal temperature. However, pV190-*CmABF3* plants showed significantly lower expression of both genes than pV190 under cold conditions (Fig. 7c and d), indicating that *CmABF3* positively regulates *CmCBF1*/2 expression in melon seedlings.

Tre content did not differ significantly between lines under normal temperature. Under cold treatment, Tre levels in both lines increased significantly, but pV190-*CmABF3* plants accumulated significantly less Tre than pV190 (Fig. 7e). Expression of *CmTPP2/5* was similar between lines under normal temperature; however, under cold conditions, pV190-*CmABF3* plants exhibited significantly lower *CmTPP2/5* expression than pV190 (Fig. 7f and g). These results confirm that CmABF3 regulates *CmTPP2/5* expression, thereby influencing Tre accumulation and *CmCBF1/2* expression under cold stress.

## Discussion

Cold stress is a major environmental constraint on crop production, severely affecting plant growth, development, and yield formation [44]. Previous work demonstrated that exogenous Tre enhanced tolerance to oxidative stress, mitigating morphological and structural cell damage, and increasing cold tolerance [25]. Although previous studies have reported that endogenous Tre plays a critical role in plant stress resistance [31], its mechanism of action remains unclear. In this study, how endogenous Tre regulates the cold response and cold tolerance of melon seedlings was examined. Cold stress markedly increased endogenous Tre levels in melon (Fig. 1a). Similar findings in other plants show that elevated endogenous Tre enhances cold tolerance [45, 46], suggesting a positive regulatory role in melons. Therefore, the relationship between Tre accumulation and cold tolerance in melon seedlings was explored.

Tre biosynthesis is catalyzed by TPS and TPP, both essential for stress defense [35, 37, 38]. TPP catalyzes the final step of Tre synthesis. In rice, *OsTPP1* and *OsTPP2* are transiently induced in both roots and shoots under cold stress, increasing TPP activity and Tre content to counter stress [46]. Overexpression of *AtTPPF* in *Arabidopsis* under drought elevates Tre, sucrose, and total soluble sugars, improving drought tolerance [40]. Likewise, overexpressing *SlTPP4* in tomato enhances glucose metabolism and salt tolerance under saline–alkali stress [47]. In the present study, transcriptomic and RT-qPCR analyses identified two cold-responsive melon genes, *CmTPP2* and *CmTPP5* (Fig. 1c–f; Fig. S1c and d). Silencing *CmTPP2* and *CmTPP5* via VIGS significantly reduced endogenous Tre content, exacerbated cold-induced damage, and diminished antioxidant capacity (Figs 2 and 3). Perhaps due to the synergistic effect of the *CmTPPs* family, and the protein modifications of TPP [48], this resulted in the continuous increase of Tre content under cold stress, but the changes in the *CmTPPs* genes family were not completely consistent. After silencing *CmTPP2/5*, the Tre content still decreased, indicating that *CmTPP2/5* positively regulates the synthesis of Tre, which also proved the above speculation. Val A is a classic and highly potent trehalase inhibitor. Its hydrolytic product, validoxylamine A, bears a strong structural resemblance to the transition state of the trehalase substrate, competitively binds to the enzyme active site, blocks the breakdown of Tre into glucose, and results in the accumulation of Tre [49]. Val A at normal concentrations is nonphytotoxic to plants, and only at high concentrations does it exert slight effects [49]. Because overexpression lines have not yet been obtained, melon seedlings were treated with Val A, which elevated endogenous Tre and significantly improved cold tolerance (Figs S3 and S4). These results indicate that *CmTPP2/5*-mediated Tre accumulation is positively associated with cold tolerance in melon seedlings. However, the specific regulatory mechanism remain to be fully defined.

Apoplastic H_2_O_2_ functions as a key signaling molecule in cold response. It can be sensed by hydrogen peroxide-induced Ca^2+^ increases 1 or/and transported by plasma membrane intrinsic protein, thereby activating pathways including CBF to enhance oxidative stress responses and chilling tolerance [5, 50, 51]. We therefore investigated whether endogenous Tre stimulates apoplastic H_2_O_2_ production and transport. Silencing *CmTPP2* and *CmTPP5* significantly impaired both apoplastic H_2_O_2_ production and transport (Fig. 4) and reduced *CmCBF1* and *CmCBF2* expression (Fig. 5). In contrast, Val A treatment increased Tre levels, promoted apoplastic H_2_O_2_ production and transport, enhanced antioxidant capacity, and upregulated *CmCBF1* and *CmCBF2* expression (Figs S4 and S5). These findings suggest that *CmTPP2/5*-mediated Tre accumulation enhances cold tolerance by activating apoplastic H_2_O_2_ signaling, facilitating its cytosolic transport, mediating redox signaling, which ultimately strengthening antioxidative defenses by partially regulating the CBF pathway (Figs S6–S8) [18, 19]. Additionally, Tre treatment partially rescued the damage induced by *CmCBF1/2* silencing (Fig. S7), suggesting that Tre may also act via the ABA-mediated ABF signaling pathway. Moreover, the ABA–ABF and CBF-dependent pathways likely exert a synergistic effect [22, 43, 52], jointly mediating the enhancement of cold tolerance in melon seedlings. Previous work also showed that OsICE1 positively regulates *OsTPP1*, increasing Tre content and improving chilling tolerance in rice [53]. As the ICE–CBF–COR pathway is a canonical cold tolerance module, Tre and CBF-dependent pathway may act in a mutually reinforcing manner, although their hierarchical relationship is not yet defined. Our previous research indicated that the interaction between CmCBF4 and H_2_O_2_ signaling triggered by exogenous Tre, which enhanced cold tolerance in melon seedlings [54]. This research illustrated that endogenous Tre mediated by CmTPP2/5 could not induced *CmCBF4* expression (Fig. 5). Exogenous Tre could function independently as a stimulant [25, 55], beyond elevating endogenous Tre levels to exert physiological effects, exogenous Tre also induces the accumulation of H_2_O_2_, NO, ABA, etc. These further mediate downstream antioxidant responses and ultimately improves plant cold tolerance. Accordingly, exogenous Tre may activate *CmCBF4* expression via the ABA–ABF pathway or other signaling cascades [54]. These indicate that exogenous and endogenous Tre have different function mechanisms in regulating the cold tolerance of melon seedlings, which still need to be studied. Furthermore, it remains to be investigated whether Tre mediated by *CmTPP2/5* can enhance the cold tolerance of melon seedlings through other pathways by activating the CmCBF1/2. We therefore examined upstream regulators of *CmTPP2* and *CmTPP5* under cold stress.

The ABF transcription factor mediated by the ABA signal plays a crucial role in the plant’s response to cold stress [22, 43]. Earlier studies identified *CmABF2* and *CmABF3* as important regulators of melon cold tolerance. These transcription factors enhance antioxidant capacity by regulating the aquaporin gene *CmPIP2;3*, thereby promoting apoplastic H_2_O_2_ production and transport [22]. In this study, promoter analysis revealed ABRE elements in *CmTPP2* and *CmTPP5* (Fig. 6a and b), which are binding sites for ABF transcription factors. Protein–DNA interaction assays confirmed that *CmABF3* binds to both promoters, activating their transcription (Fig. 6c–g). Silencing *CmABF3* reduced *CmTPP2/5* and *CmCBF1*/2 expression and lowered Tre content (Fig. 7). These results indicate that cold-induced *CmABF3* enhances cold tolerance by activating *CmTPP2/5* expression, thereby promoting Tre synthesis.

There are few reports on the mechanism of endogenous Tre in regulating plant stress response and resistance. The existing reports indicate that accumulated Tre in plant has been described as a sugar signal, osmoprotectant, or regulators of other sugars accumulation and antioxidation to enhanced cold tolerance [45]. Additionally, Tre has been reported to stabilize membrane lipids and protein stability under stress conditions, but the specific mechanism remains to be explored [31]. This study suggest Tre also acts by activating apoplastic H_2_O_2_ signaling. Whether apoplastic H_2_O_2_ reciprocally regulates Tre synthesis remains to be determined. The results of this study provide at least a new ideas for subsequent research on the mechanism of Tre’s action. ABF transcription factors as a key mediators of ABA signaling—a central regulator of stress responses, including cold tolerance [52, 54, 56]—and previous work has implicated ABA in melon cold tolerance [25, 43]. Whether there is a correlation between the ABA and Tre signals requires further investigation. Additionally, in the preliminary experiments, we made multiple attempts but have not yet successfully obtained genetically transformed materials of melon *CmTPP2*, *CmTPP5*, and *CmABF3*. Therefore, we employed the cucurbit-specific transient silencing vector pV190 to verify the functions of these genes in this study [33, 57, 58] . Once transgenic materials for these genes are obtained in later stages, it will be necessary to further investigate their functions.

## Conclusion

In conclusion, a model (Fig. 8) for the regulation of melon cold tolerance is proposed. Cold stress induces CmABF3, which binds to ABRE elements in *CmTPP2* and *CmTPP5* promoters, activating their transcription and increasing Tre synthesis. Elevated Tre stimulates apoplastic H_2_O_2_ production and transport into the cytoplasm, and coordination with the CBF-dependent pathway to improve cold tolerance in melon seedlings.

**Figure 8:**
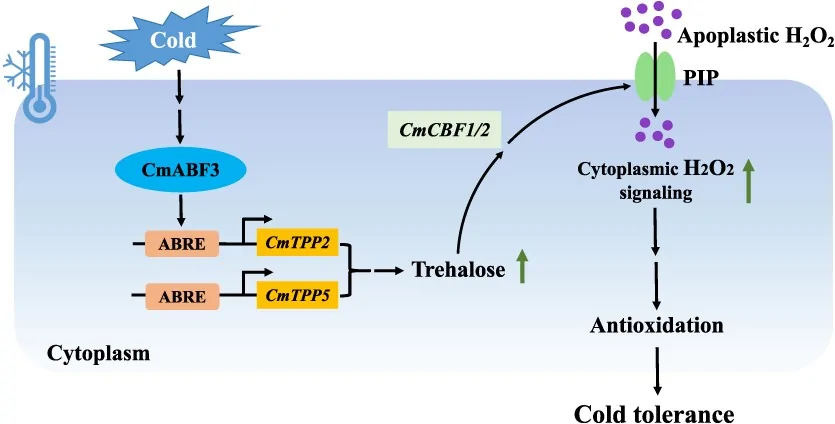
Proposed model of CmABF3-*CmTPP2/5*-induced Tre enhancing cold tolerance in melon seedlings. Cold-induced *CmABF3* binds to the *CmTPP2/5* promoter and activates their expression, increasing endogenous Tre levels. Elevated Tre enhances apoplastic H_2_O_2_ production and transport, enhancing downstream antioxidation and improving cold tolerance in melon seedlings. This pathway may be associated with the CBF-dependent pathway induced by Tre.

## Materials and methods

### Plant materials and treatments

The cold-sensitive oriental melon (*Cucumis melo* L.) variety ‘IVF004’ was used as experimental material. Germinated seeds were sown in 50-cell trays containing a substrate mixture of peat, vermiculite, and perlite (volume ratio, 2:1:1). Seedlings were grown in an artificial climate chamber. After the emergence of the second true leaf, seedlings were transplanted into 7.0 × 7.0 cm pots. Growth conditions were maintained at 28°C/18°C (day/night), with a 12 h photoperiod (photosynthetic photon flux density, 500 μmol·m^−2^·s^−1^) and 65% ± 5% relative humidity. Seedlings with fully expanded third true leaves were used for experiments.

VIGS was employed to generate *CmTPP2*, *CmTPP5*, *CmABF3*, *CmCBF1*, and *CmCBF2*-silenced lines. Following the method of Xu *et al.* [33], ~300 bp fragments from the full-length coding sequence (CDS) of *CmPDS*, *CmTPP2*, *CmTPP5*, *CmABF3*, *CmCBF1*, and *CmCBF2* were inserted into the pV190 vector to generate pV190-*CmPDS*, pV190-*CmTPP2*, pV190-*CmTPP5*, pV190-*CmABF3*, pV190-*CmCBF1*, and pV190-*CmCBF2* constructs. Recombinant vectors were transformed into *Agrobacterium tumefaciens* strain GV3101 by freeze–thaw transformation. Melon seedlings with fully expanded cotyledons were infiltrated with the constructs, incubated in the dark for 48 h, and then transferred to standard growth conditions (28°C/18°C, day/night). Seedlings with fully expanded third true leaves were used for experiments. Primers used are listed in Table S1.

To examine the effect of cold stress on Tre content and *CmTPPs* expression, seedlings were divided into two groups: one maintained under control conditions (28°C/18°C, day/night) and the other exposed to cold conditions (15°C/6°C, day/night). Tre content in the third true leaf was measured at 0, 3, 6, 12, 15, and 24 h after cold treatment. After 18 h of treatment, when the leaves begin to show slight wilting, the third leaves were collected from both treatment conditions for transcriptome sequencing. The third true leaves were collected at 0, 6, 12, 15, 18, and 24 h from both treatments for *CmTPPs* expression analysis by RT-qPCR. Specific expression of *CmTPPs* were also analyzed in different plant tissue under normal conditions. Primers used are listed in Table S1.

To determine the role of *CmTPP2/5* in cold tolerance, pV190, pV190-*CmTPP2*, and pV190-*CmTPP5* lines were subjected to control (28°C/18°C, day/night) and cold (15°C/6°C, day/night) conditions. The H_2_O_2_-specific dyes AUR and AR with distinct membrane permeabilities were used to measure apoplastic and cytoplasmic H_2_O_2_ levels, respectively [22]. As cold stress can damage cell membranes and affect probe accuracy, H_2_O_2_ levels were measured prior to temperature treatment. REC, MDA content, FRAP, antioxidase (SOD, APX, and GR) activities, and the *CmCBFs* expression were determined after 24 h of temperature treatment. Seedling phenotypes were photographed.

To determine the role of *CmCBF1/2* in cold tolerance, pV190, pV190-*CmCBF1*, and pV190-*CmCBF2* lines were presprayed with H_2_O or 10 mM Tre, 12 h later, the plants were subjected to normal (28°C/18°C, day/night) and cold (15°C/6°C, day/night) conditions. The REC, MDA content, and *CmABF3* expression were determined after 24 h of temperature treatment. Seedling phenotypes were photographed. Additionally, to explore whether Tre can partially compensate for the silencing of *CBF1/2* genes on apoplastic and cytoplasmic H_2_O_2_ levels, respectively, melon seedlings were sprayed with 10 mM Tre [25], then kept at room temperature for 12 h before sampling to determine apoplastic and cytoplasmic H_2_O_2_ levels using AUR and AR.

To further assess whether increased Tre levels improve cold tolerance, seedlings were sprayed with the trehalase inhibitor Val A at 0, 75, or 150 μM (selected based on preliminary experiments). After 12 h at control temperature, apoplastic and cytoplasmic H_2_O_2_ levels were measured. Plants were then divided into control (28°C/18°C, day/night) and cold (15°C/6°C, day/night) groups. After 24 h of cold treatment, the third true leaf was sampled for REC, MDA, FRAP, the antioxidase (SOD, APX, GR) activities assays, the Tre content, and *CmCBF1/2* expression, and seedling phenotypes were photographed.

### Phylogenetic analysis of the *CmTPPs* gene family

To identify the *CmTPPs* gene family in melon, the Cucurbitaceae genome database was searched using ‘*CmTPPs*’ as a keyword. The amino acid sequences of *Arabidopsis* TPP family members were also used as queries for BLASTP searches in the same databases. Sequences from both approaches were aligned using DNAMAN 6.0, and those lacking the conserved family domain were removed. A phylogenetic tree was constructed in MEGA 11.

### Chromosomal localization of *CmTPPs*

The chromosome localization of the *CmTPPs* gene family was analyzed and visualized using the TBtools software.

### Measurement of relative electrical conductivity and malondialdehyde content

REC was determined according to the methods of Zhou and Leul [57]. MDA content was measured using the thiobarbituric acid (TBA) method. The leaf sample was ground to a powder in liquid nitrogen. A 1.7 ml of 10% trichloroacetic acid was added, vortexed for 1 min, and incubated at room temperature for 10 min. The homogenate was centrifuged at 15 000 rpm for 10 min. A 0.9 ml aliquot of the supernatant was mixed with 0.9 ml of 0.6% TBA solution. The mixture was heated in a boiling water bath for 15 min, cooled, and the supernatant absorbance was measured at 450, 532, and 600 nm. MDA content was calculated using the formula described by Hodges *et al.* [58].

### Determination of total antioxidant capacity (ferric-reducing antioxidant power)

FRAP was assayed following Benzie and Strain [59] and Xu *et al.* [60]. The sample was ground in liquid nitrogen. Powder was extracted with 1.6 ml methanol. After centrifugation at 14 000 rpm for 10 min, 100 μl of supernatant was mixed with 300 μl distilled water and 3 ml of TPTZ working solution. The mixture was incubated at 37°C for 10 min, and absorbance was measured at 593 nm.

### Assay of antioxidase activities

The leaf sample was ground to a powder in liquid nitrogen. A 50 mg polyvinylpyrrolidone and a 1.5 ml of ice-cold 0.1 M sodium phosphate buffer [pH 7.5, containing 1 mM ethylenediaminetetraacetic acid (EDTA)] were added, and grinding continued until homogenized. The homogenate was centrifuged at 4°C and 15 000 rpm for 10 min. The supernatant was used for enzyme assays.

SOD activity was determined according to the method of Giannopolitis and Ries [61]: 3 ml of supernatant was added with 30 μl of reaction mixture, and then exposed to 4000 lux light for ~10 min. The absorbance at OD560 was measured.

APX activity was determined as described by Noctor *et al.* [62]: 50 μl of supernatant was added with 50 μl of 10 mM AsA, 890 μl of 0.1 M sodium phosphate buffer (containing 1 mM EDTA, pH 7.5), and 10 μl of 20 mM H_2_O_2_ was added to start the reaction, and the decrease in A290 within 1–2 min was measured.

GR activity was determined as described by Noctor *et al.* [62]: 100 μl of supernatant was added with 10 μl of 10 mM NADPH, 880 μl of 0.1 M sodium phosphate buffer, pH 7.5 (containing 1 mM EDTA), mixed and 10 μl of 50 mM oxidized glutathione (GSSG) was added to start the reaction. The decrease in A340 within 2–3 min was recorded.

### Hydrogen peroxide fluorescence signal detection

The H_2_O_2_ in the cytoplasm and apoplast was visualized using the specific fluorescent probes AUR and AR, respectively, under a confocal laser scanning microscope. Fluorescence emission was detected at 585–610 nm upon excitation at 525 nm. Fluorescence signal intensity was quantified using ImageJ software. Leaf samples from different treatments were immediately immersed in phosphate buffered saline (PBS) for 15–30 min. Samples were then incubated in solutions containing AUR or AR for 15–30 min prior to observation [22]. AUR + AR represents the total levels of H_2_O_2_ in apoplast and cytoplasm, while AUR/AR represents the ratio of H_2_O_2_ levels in the apoplast to those in the cytoplasm. From the above, it can be concluded the amount of apoplast H_2_O_2_ that is transported into the cytoplasm under the conditions of AUR + AR being relatively constant.

### Trehalose content determination

Tre content was measured using the assay kit method (AKSU005C, Boxbio Science & Technology Co., Ltd, Beijing, China) according to the manufacturer’s instructions.

### RNA and DNA extraction

The RNA in the leaves of melon seedlings was extracted using the RNA extraction kit (CWBIO, Beijing, China). The extracted RNA was reverse transcribed into cDNA using PrimeScript™ RT Master Mix (Takara, Dalian, China) for subsequent CDS cloning and RT-qPCR. The DNA from the melon leaves was extracted using the DNA extraction kit (Tiangen, Beijing, China) for the cloning of the promoter fragment.

### Quantitative real-time polymerase chain reaction

RT-qPCR was performed using PerfectStart® Green qPCR Super Mix (TransGen Biotech, Beijing, China) according to the manufacturer’s protocol. Gene expression levels were calculated using the comparative threshold cycle (2^–ΔΔCt^) method. The specific primers are listed in Table S1.

### Yeast one-hybrid assay

Promoter fragments of *CmTPP2* and *CmTPP5* were cloned into the pAbAi vector. The cDNA sequences of *CmABF2* and *CmABF3* were cloned into the pGADT7 vector. The *CmTPP2* and *CmTPP5* promoter-pAbAi constructs were linearized and transformed into Y1H Gold yeast strain using a yeast transformation kit (Takara, Dalian, China). The recombinant pGADT7-CmABF2/3 plasmids were individually transformed into Y1H Gold strains harboring the respective target promoter. Transformants were plated on SD/−Leu solid medium (Takara, Dalian, China) and incubated at 30°C for 3–5 days. Protein–DNA interactions were assessed based on yeast growth. Primers used are listed in Table S1.

### β-Glucuronidase activity assay

The CDS of *CmABF3* was cloned into the pRI101 as an effector vector. Promoter fragments of *CmTPP2* and *CmTPP5* were cloned into the pBI101-GUS as a reporter vector. Recombinant vectors and empty vector controls were transformed into *A. tumefaciens* strain GV3101. Appropriate seedling age tobacco (*Nicotiana benthamiana*) plants were infiltrated using a 1-ml syringe with a 1:1 mixture of reporter and effector *Agrobacterium* suspensions. The tobacco leaves were cultured in the dark for 24 h and under low light for 2 days for sampling. The total tobacco protein was extracted for GUS activity determination [63]. Primers used are listed in Table S1.

### Luciferase fluorescence intensity assay

Promoter fragments of *CmTPP2* and *CmTPP5* were cloned into pGreenII 0800-LUC as a reporter vector. The CDS of *CmABF3* was cloned into pCAMBIA1300-35S-GFP as an effector vector. The reporter and effector gene vector infection solution were mixed in equal proportion and co-expressed in tobacco leaves according to the specified combination. After dark culture for 24 h and weak light culture for 2 days, the leaves were cut and evenly smeared with D-Luciferin potassium salt (MCE, USA) on the back of the leaves. After dark incubation for 10 min, the LUC signal was detected using a live cell fluorescence imaging system. Primers used are listed in Table S1.

### Electrophoretic mobility shift assay

The full-length coding sequence of CmABF3 was cloned and inserted into the pET-32a vector. The subsequent protein induction and purification procedures were performed following a previously established protocol with minor adjustments. Biotin-labeled probes targeting the ABRE motifs within the *CmTPP2* and *CmTPP5* promoter regions, along with corresponding mutant probes, were synthesized by Sangon Biotech (Shanghai, China). EMSA experiments were carried out using the EMSA Kit (Beyotime Biotechnology) according to the manufacturer’s instructions. The primers used for vector construction and probe design are listed in Table S1.

### Statistical analysis

All experiments were performed with at least three biological replicates. Data were analyzed in SPSS 26.0 using Duncan’s multiple range test. Statistical significance was set at *P* <0 .05 (denoted by different letters or *) and *P* <0 .01 (**).

## Supplementary Material

Web_Material_uhag233

## Data Availability

All the supplementary data relevant to this study are presented online.
