# Supplementary material for: CmABF3-CmTPP2/5 modulates trehalose-mediated hydrogen peroxide signaling and CBF-dependent pathway to enhance cold tolerance in melon seedlings
Source: Hortic Res. 2026 Jun 8;13(11):uhag233. doi: 10.1093/hr/uhag233 (PMC13723084; doi:10.1093/hr/uhag233)
Supplement: Supplementary file 1 [file web_material_uhag233.zip › Supplementary_Figures_uhag233.pdf]

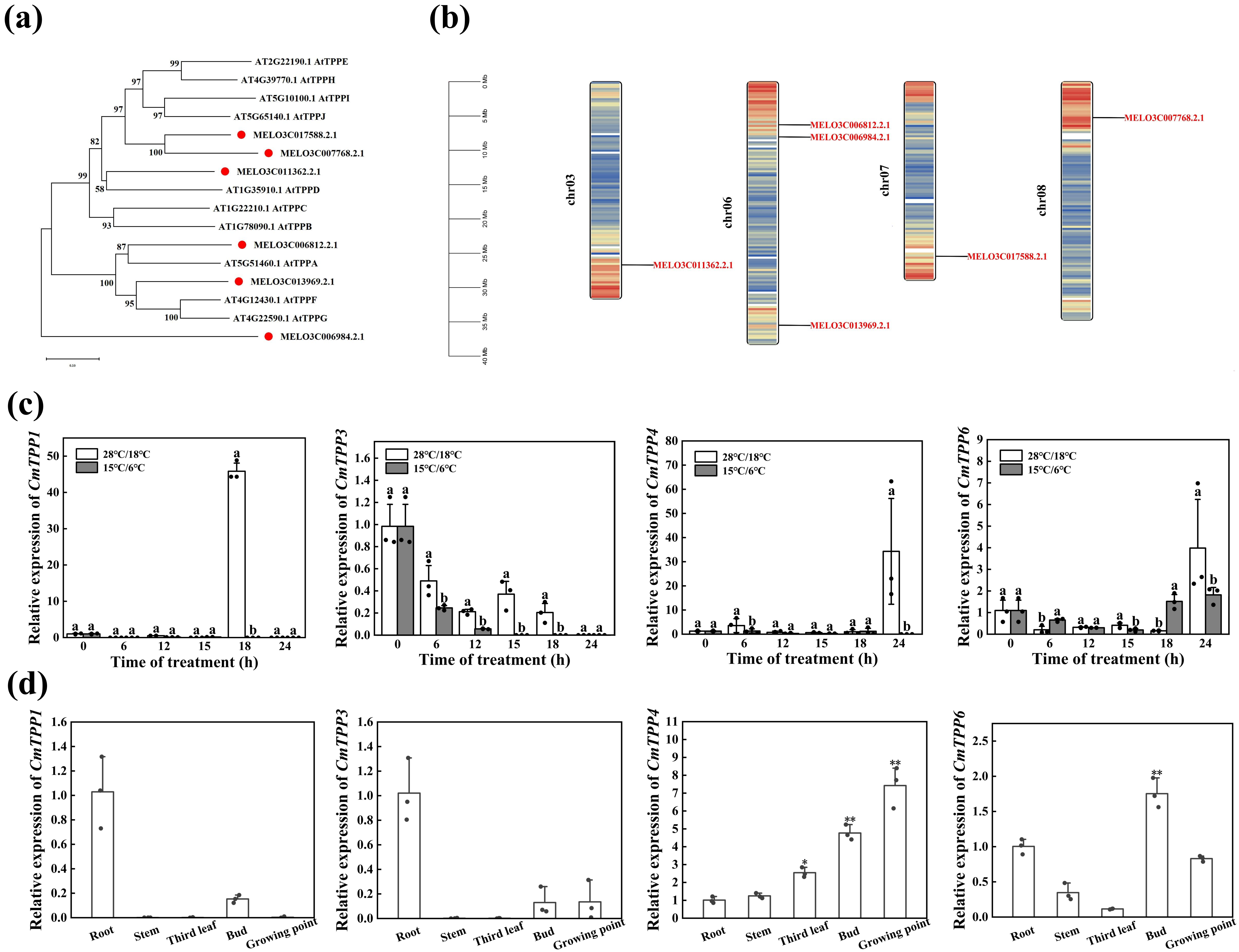

(a)
AT2G22190.1 AtTPPE
AT4G39770.1 AtTPPH
AT5G10100.1 AtTPPI
AT5G65140.1 AtTPPJ
MELO3C017588.2.1
MELO3C007768.2.1
MELO3C011362.2.1
AT1G35910.1 AtTPPD
AT1G22210.1 AtTPPC
AT1G78090.1 AtTPPB
MELO3C006812.2.1
AT5G51460.1 AtTPPA
MELO3C013969.2.1
AT4G12430.1 AtTPPF
AT4G22590.1 AtTPPG
MELO3C006984.2.1
(b)
0 Mb
5 Mb
10 Mb
15 Mb
20 Mb
25 Mb
30 Mb
35 Mb
40 Mb
chr03
chr06
chr07
chr08
MELO3C011362.2.1
MELO3C006812.2.1
MELO3C006984.2.1
MELO3C013969.2.1
MELO3C017588.2.1
MELO3C007768.2.1
(c)
Relative expression of CmTPP1
Relative expression of CmTPP3
Relative expression of CmTPP4
Relative expression of CmTPP6
28°C/18°C
15°C/6°C
Time of treatment (h)
(d)
Relative expression of CmTPP1
Relative expression of CmTPP3
Relative expression of CmTPP4
Relative expression of CmTPP6
Root
Stem
Third leaf
Bud
Growing point

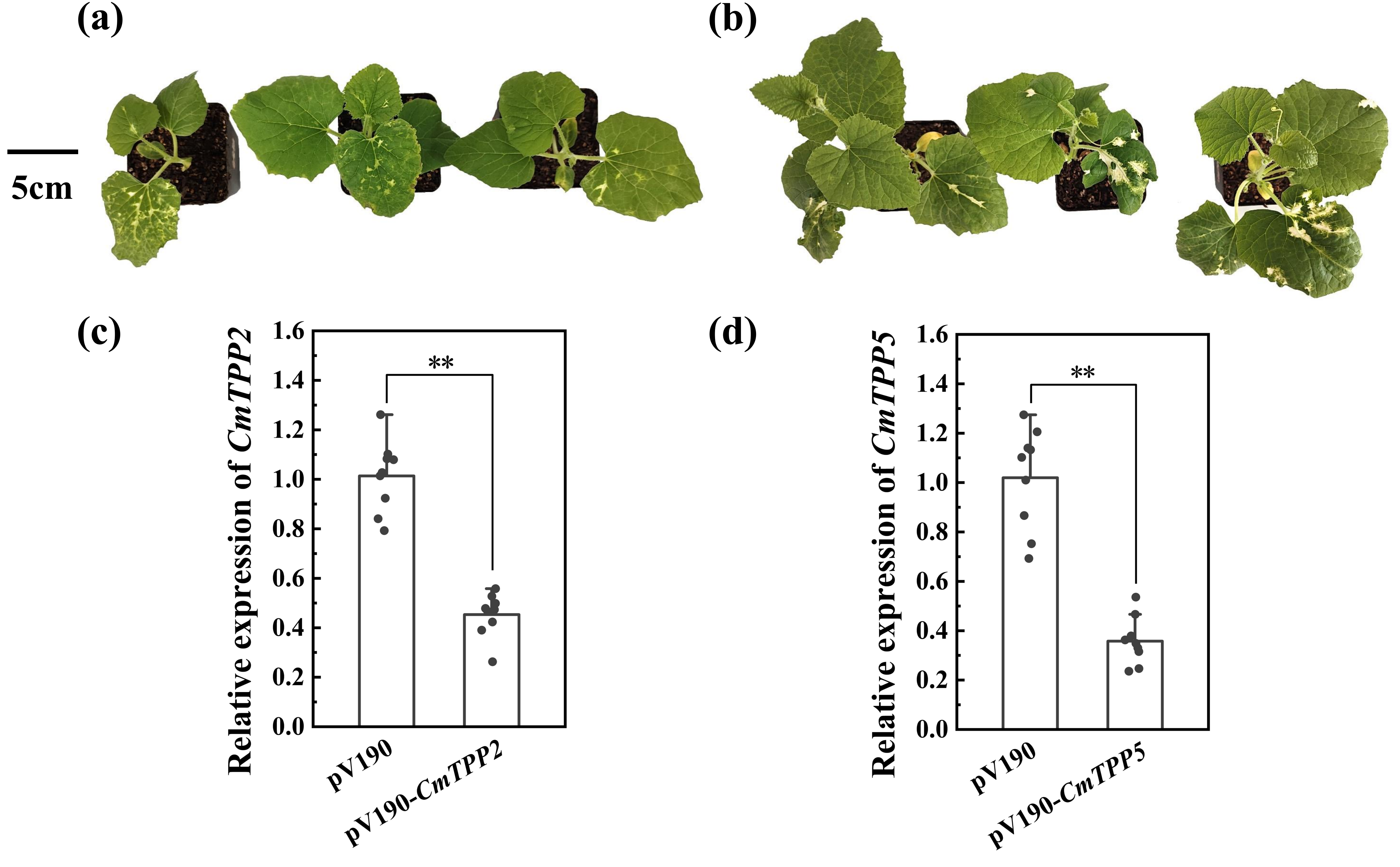

(a)
(b)
5cm
(c)
(d)
Relative expression of CmTPP2
Relative expression of CmTPP5
1.6
1.4
1.2
1.0
0.8
0.6
0.4
0.2
0.0
**
pV190
pV190-CmTPP2
pV190-CmTPP5

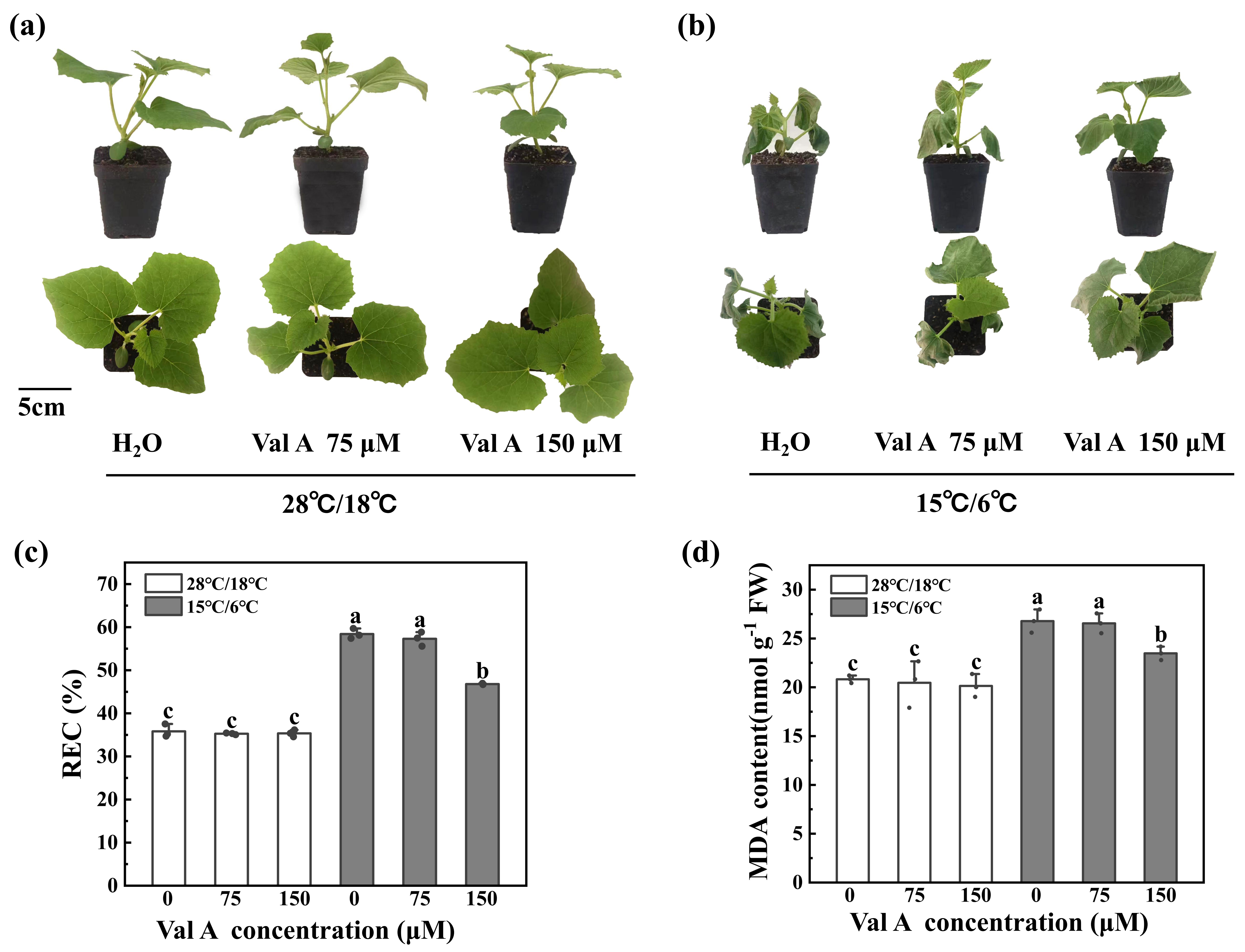

(a)
(b)
5cm
H2O
Val A 75 μM
Val A 150 μM
28℃/18℃
H2O
Val A 75 μM
Val A 150 μM
15℃/6℃
(c)
28°C/18°C
15°C/6°C
REC (%)
Val A concentration (μM)
(d)
28°C/18°C
15°C/6°C
MDA content(nmol g-1 FW)
Val A concentration (μM)

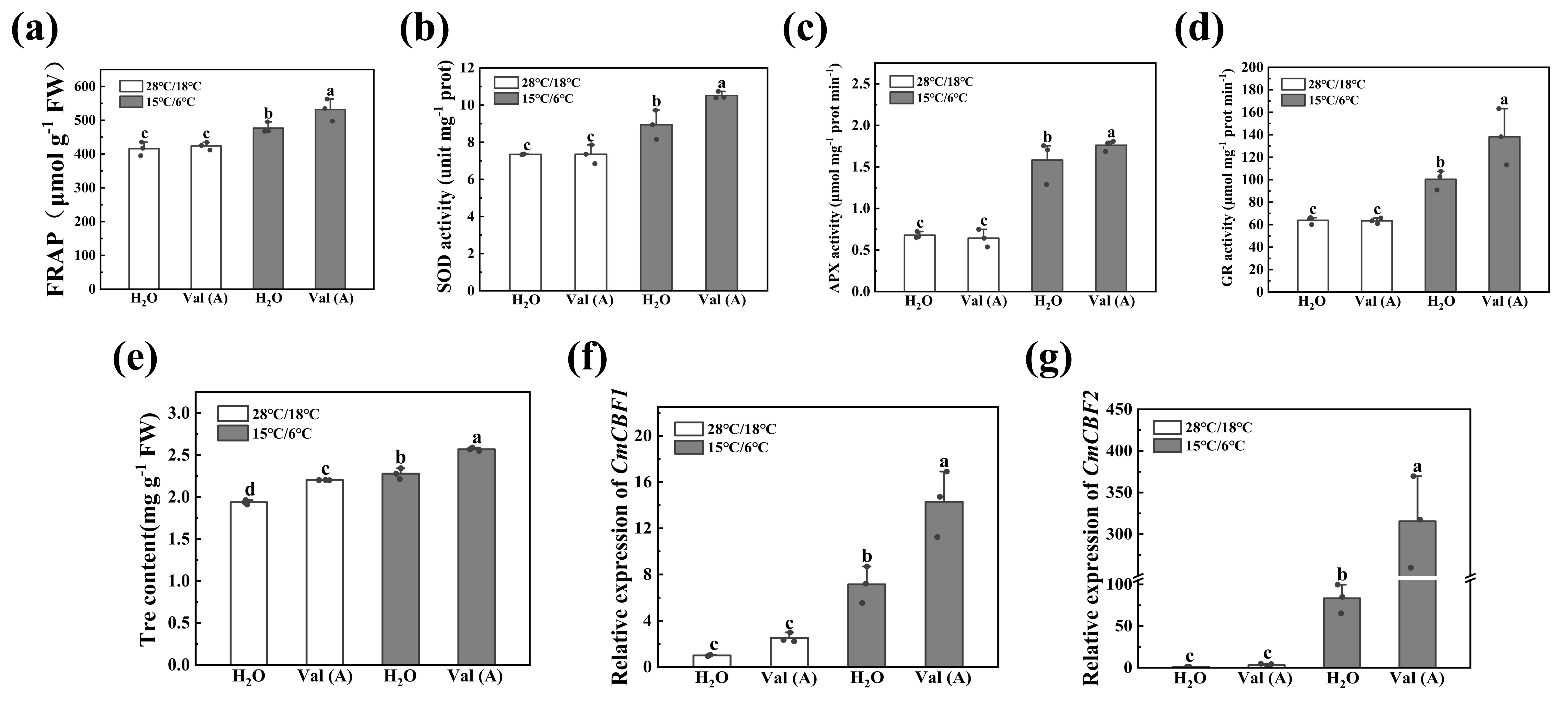

(a)
FRAP（μmol g-1 FW）
28°C/18°C
15°C/6°C
H2O
Val (A)
(b)
SOD activity (unit mg-1 prot)
(c)
APX activity (μmol mg-1 prot min-1)
(d)
GR activity (μmol mg-1 prot min-1)
(e)
Tre content(mg g-1 FW)
(f)
Relative expression of CmCBF1
(g)
Relative expression of CmCBF2

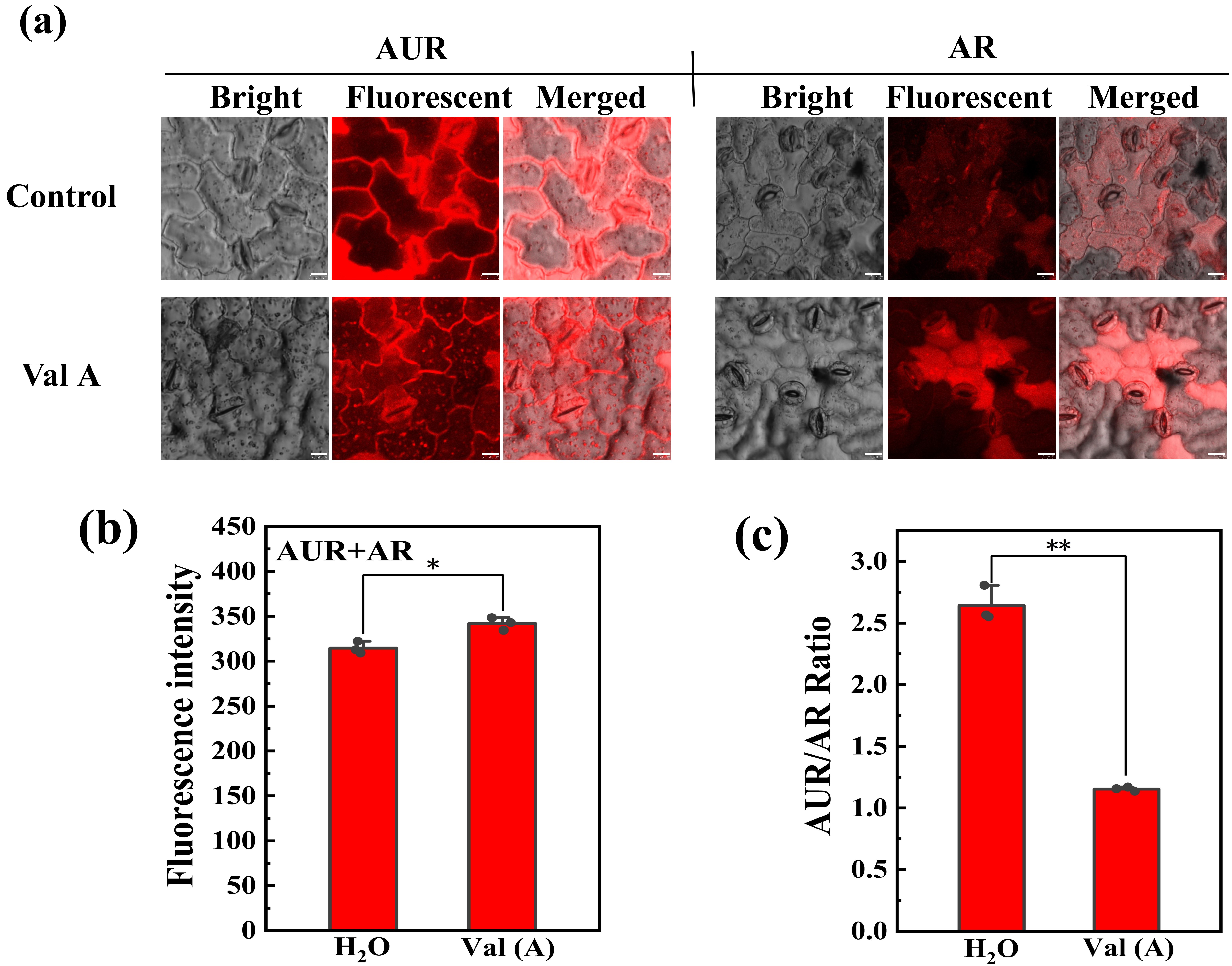

(a)
AUR
AR
Bright
Fluorescent
Merged
Control
Val A
(b)
AUR+AR
*
Fluorescence intensity
450
400
350
300
250
200
150
100
50
0
H2O
Val (A)
(c)
**
AUR/AR Ratio
3.0
2.5
2.0
1.5
1.0
0.5
0.0

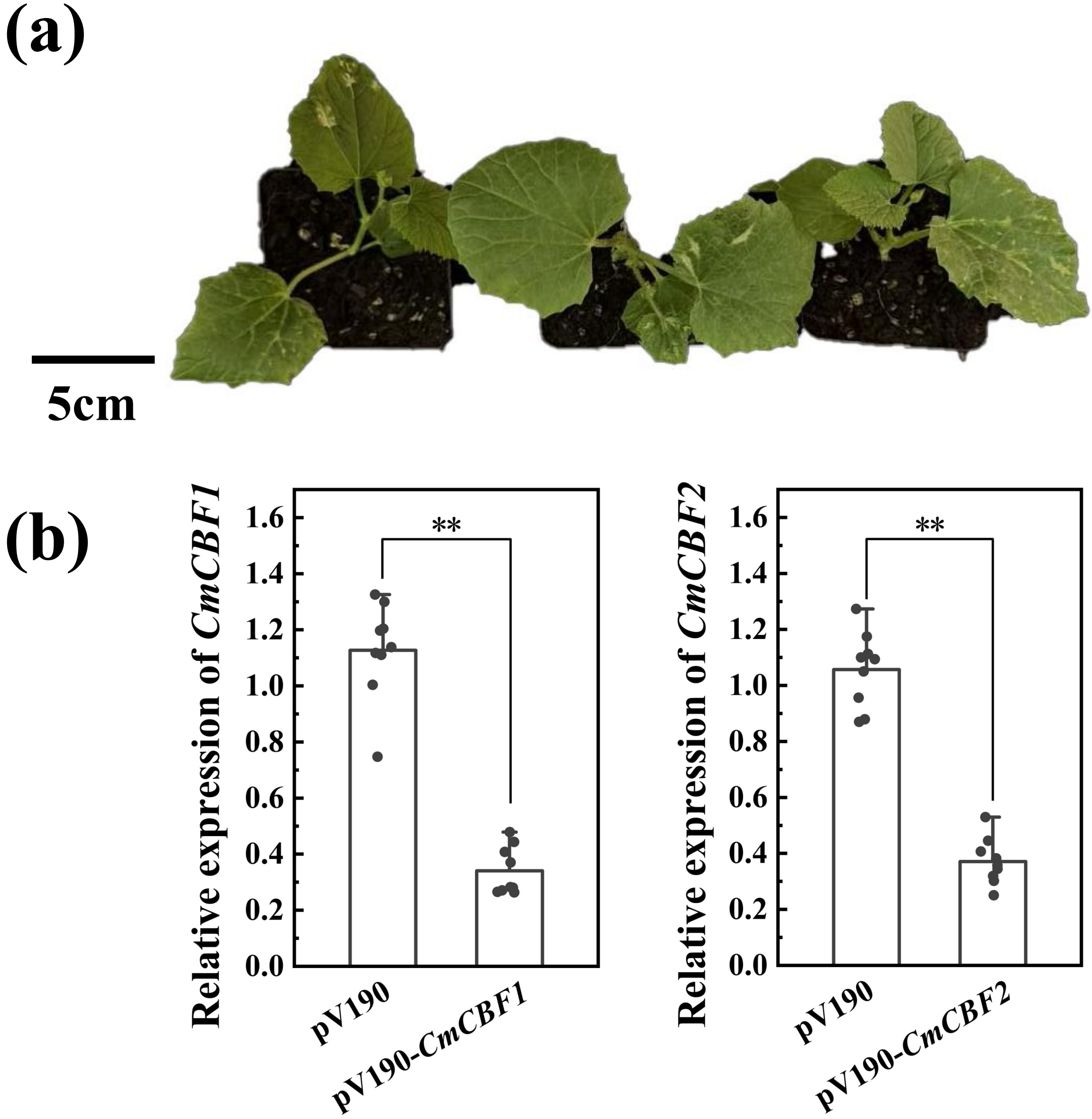
(a)
5cm
(b)
Relative expression of CmCBF1
1.6
1.4
1.2
1.0
0.8
0.6
0.4
0.2
0.0
**
pV190
pV190-CmCBF1
Relative expression of CmCBF2
**
pV190
pV190-CmCBF2

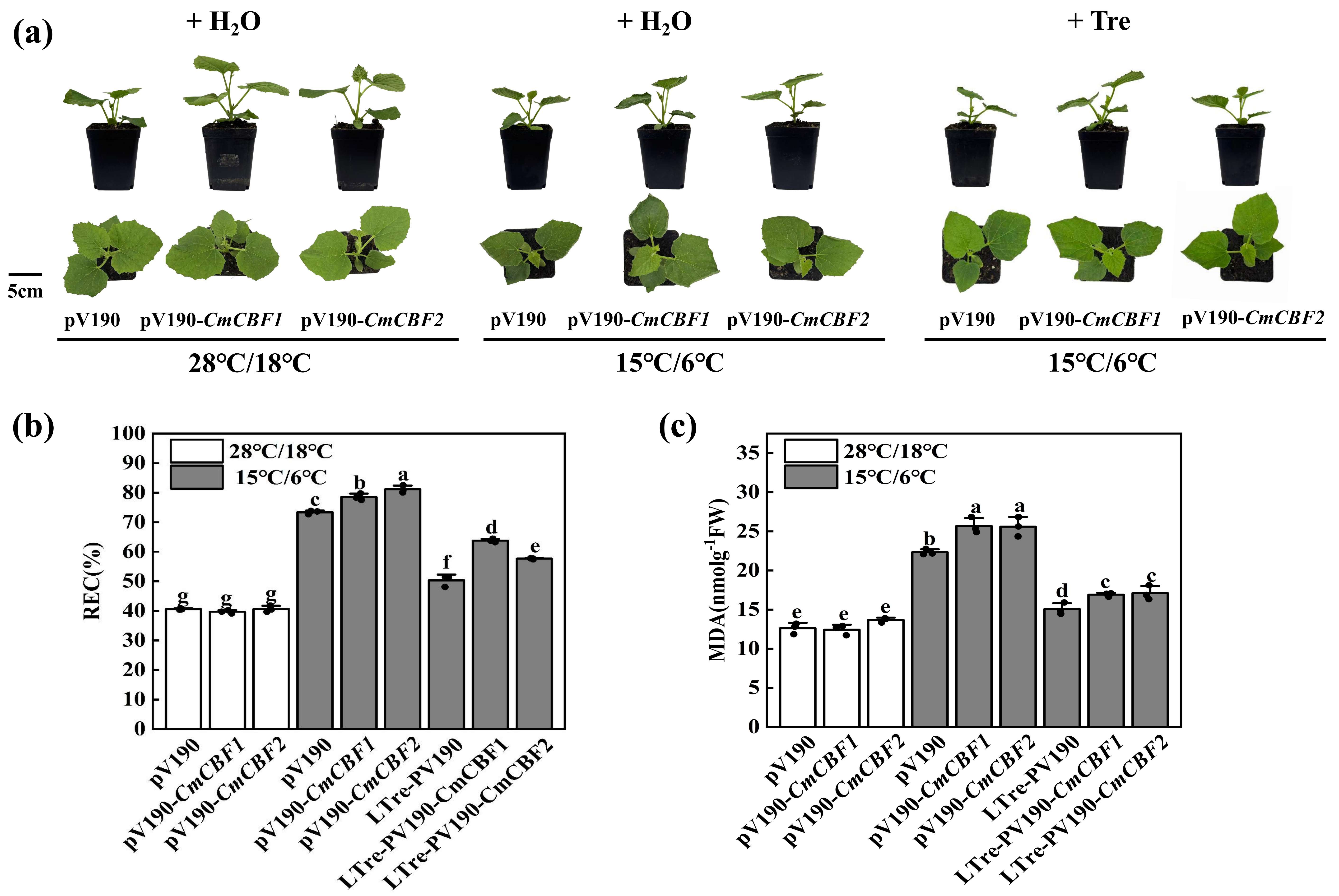

(a)
+ H2O
+ H2O
+ Tre
5cm
pV190
pV190-CmCBF1
pV190-CmCBF2
pV190
pV190-CmCBF1
pV190-CmCBF2
pV190
pV190-CmCBF1
pV190-CmCBF2
28°C/18°C
15°C/6°C
15°C/6°C
(b)
REC(%)
28°C/18°C
15°C/6°C
pV190
pV190-CmCBF1
pV190-CmCBF2
pV190
pV190-CmCBF1
pV190-CmCBF2
LTre-PV190
LTre-PV190-CmCBF1
LTre-PV190-CmCBF2
(c)
MDA(nmolg-1FW)
28°C/18°C
15°C/6°C
pV190
pV190-CmCBF1
pV190-CmCBF2
pV190
pV190-CmCBF1
pV190-CmCBF2
LTre-PV190
LTre-PV190-CmCBF1
LTre-PV190-CmCBF2

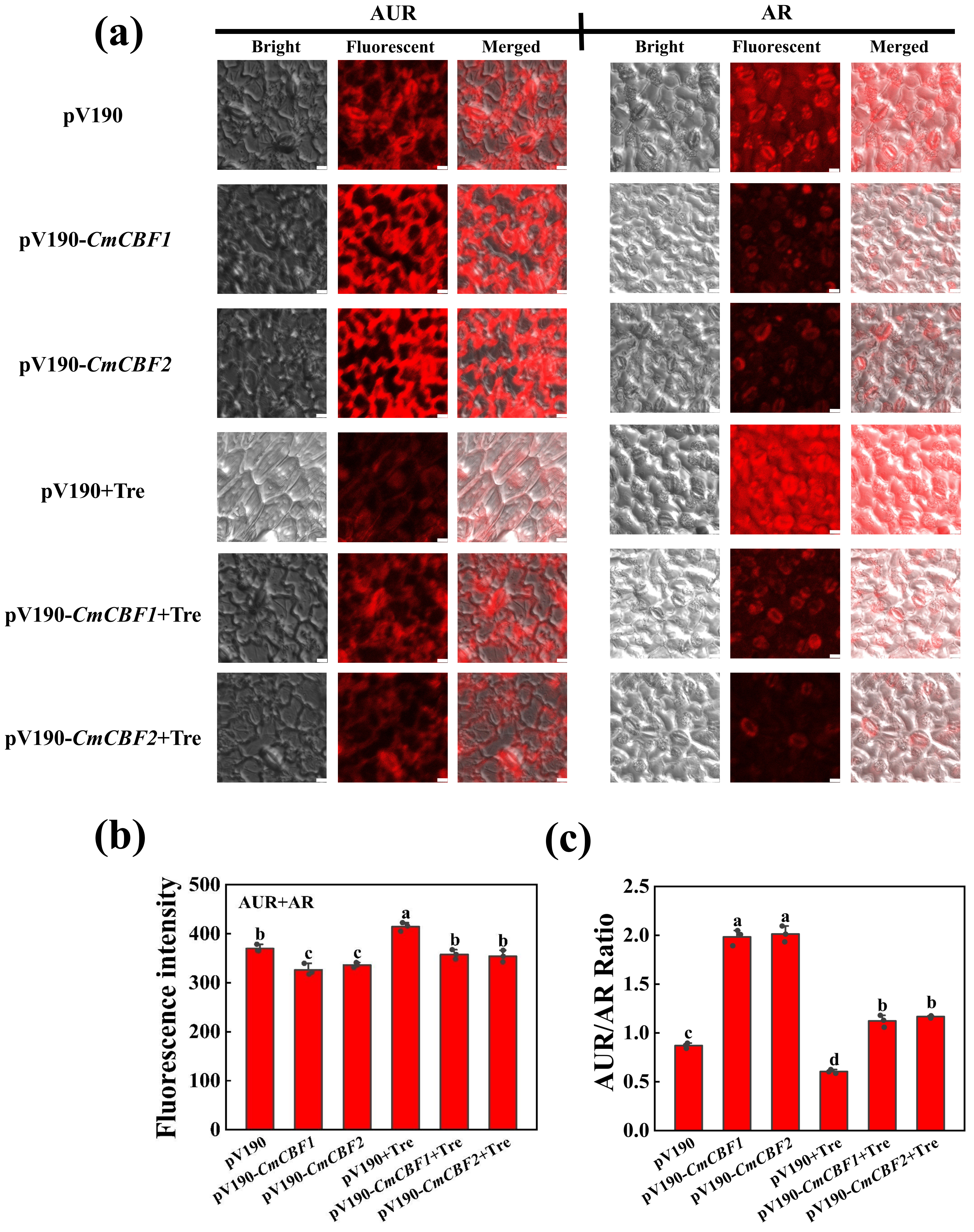
(a)
AUR
AR
Bright
Fluorescent
Merged
pV190
pV190-CmCBF1
pV190-CmCBF2
pV190+Tre
pV190-CmCBF1+Tre
pV190-CmCBF2+Tre
(b)
Fluorescence intensity
AUR+AR
0
100
200
300
400
500
a
b
c
(c)
AUR/AR Ratio
0.0
0.5
1.0
1.5
2.0
2.5
d
